# Complications following surgical treatment of posterior malleolar fractures: an analysis of 300 cases

**DOI:** 10.1007/s00402-022-04536-9

**Published:** 2022-07-18

**Authors:** Annika Pauline Neumann, Livia Kroker, Franziska Beyer, Stefan Rammelt

**Affiliations:** University Center of Orthopaedics,Trauma and Plastic Surgery, University Hospital Carl Gustav Carus at TU Dresden, Fetscherstrasse 74, 01307 Dresden, Germany

**Keywords:** Ankle, Malleolar fracture, Posterior tibia, Complication, Revision

## Abstract

**Aims:**

The treatment of ankle fractures and fracture-dislocations involving the posterior malleolus (PM) has undergone considerable changes over the past decade. The aim of our study was to identify risk factors related to the occurrence of complications in surgically treated ankle fractures with PM involvement.

**Patients and methods:**

We retrospectively analyzed 300 patients at a mean age of 57 years with 300 ankle fractures involving the PM treated surgically at our institution over a 12-year period. The following relevant comorbidities were noted: arterial hypertension (43.7%; *n* = 131), diabetes mellitus (DM) (14.0%; *n* = 42), thereof insulin-dependent (3.7%; *n* = 11), peripheral vascular disease (0.7%; *n* = 2), osteoporosis (12.0%; *n* = 36), dementia (1.0%; *n* = 3), and rheumatoid arthritis (2.0%; *n* = 6). Furthermore, nicotine consumption was recorded in 7.3% (*n* = 22) and alcohol abuse in 4.0% (*n* = 12).

**Results:**

Complications occurred in 41 patients (13.7%). A total of 20 (6.7%) revision surgeries had to be performed. Patients with DM (*p* < 0.001), peripheral vascular disease (*p* = 0.003) and arterial hypertension (*p* = 0.001) had a significantly increased risk of delayed wound healing. Alcohol abuse was associated with a significantly higher overall complication rate (OR 3.40; 95% CI 0.97–11.83; *p* = 0.043), increased rates of wound healing problems (OR 11.32; 95% CI 1.94–65.60; *p* = 0.001) and malalignment requiring revision (*p* = 0.033). The presence of an open fracture was associated with an increased rate of infection and wound necrosis requiring revision (OR 14.25; 95% CI 2.39–84.84; *p* < 0.001). Multivariate analysis identified BMI (*p* = 0.028), insulin-dependent DM (*p* = 0.003), and staged fixation (*p* = 0.043) as independent risk factors for delayed wound healing. Compared to the traditional lateral approach, using the posterolateral approach for fibular fixation did not lead to increased complication rates.

**Conclusions:**

Significant risk factors for the occurrence of complications following PM fracture treatment were identified. An individually tailored treatment regimen that incorporates all risk factors is important for a good outcome.

## Introduction

Ankle fractures are among the most common injuries to a weight-bearing joint of the lower extremity [1]. Involvement of the posterior malleolus (PM) occurs in up to 50% of all ankle fractures and has been traditionally fraught with inferior outcome [2, 3]. Anatomic reduction of the incisura and articular surface combined with low complication rates is the goal of treatment [4].

The treatment of ankle fractures and fracture-dislocations involving the PM has undergone considerable changes over the past decade [5, 6]. With an individualized approach to the fracture morphology as assessed with preoperative computed tomography (CT) scanning, the historically poor outcomes of trimalleolar ankle fractures could be improved substantially [7–10]. However, although the indications to surgery and techniques of reduction and fixation have been refined, there is still controversy with respect to the individual approach and concerns have been raised about possible complications with the increased use of posterior approaches [11–13]. Several studies have demonstrated that complications have a negative impact on outcome of ankle fracture treatment [12, 14–16].

The aim of our study was to identify risk factors related to the occurrence of complications in surgically treated ankle fractures involving the PM.

## Materials and methods

A retrospective chart review was performed of all patients with a malleolar fracture involving the PM treated operatively at our institution between 2003 and 2015. Exclusion criteria were age under 18 years, pathological fractures, polytraumatized patients and concomitant fractures of the same limb.

This left 300 patients (207 female and 93 male) with 300 fractures and an average age of 56.8 years (range 18–92) at the time of injury for analysis. The BMI was available for 166 patients. It averaged 27.12 kg/m^2^ (range 17.7–51.6; SD 6.05).

Nineteen patients (6.3%) had bimalleolar fractures, 201 (67%) trimalleolar fractures and 78 (26%) quadrimalleolar fractures [17]. Of the latter, 32 (41%) were trimalleolar fractures with an additional anterior tibial tubercule (Tillaux-Chaput) fragment, 43 (55.1%) were trimalleolar fractures with an additional anterior fibular tubercle (Wagstaffe-LeFort) fragment (quadrimalleolar equivalent) and 3 (3.9%) were trimalleolar fractures with additional tubercule de Tillaux-Chaput and Wagstaffe-LeFort fragments. Furthermore, one patient (0.3%) presented an isolated fracture of the PM and another patient (0.3%) sustained a combined fracture of the fibula, the PM, the tubercule de Tillaux-Chaput and Wagstaffe-LeFort fragment. Ten fractures (3.3%) were open. Fracture-dislocations were seen in 107 (35.7%) cases.

One-stage internal fixation was performed in 156 fractures (52%) and staged treatment with primary external fixation and secondary internal fixation in 143 (47.7%). In one patient (0.3%), the fracture was treated with external fixation only. Open reduction and direct posterior fixation of the PM fragment was performed in 122 patients (40.6%). Of these, 89 (29.7%) were fixed with a plate and 33 (11%) with posterior-to-anterior (PA) lag screws. A total of 109 PM fractures (36.3%) were treated via a posterolateral, 7 (2.3%) via a posteromedial and 6 (2%) via a medial approach. Thirty-eight (12.7%) PM fractures were fixed indirectly with anterior-to-posterior (AP) lag screws through a small anterior approach, mostly with transfibular control of reduction [6]. In 140 patients (46.7%) with Bartoníček [18] type I or non-displaced type II and III fractures, the PM fragment was not fixed.

In 225 of the 293 fibular fractures (76.8%), a lateral approach was used for open reduction and internal fixation. In 65 cases (22.2%), the fibula and PM fragment were fixed via a posterolateral approach. In 3 cases (1.0%), a percutaneous screw fixation was performed to the lateral malleolus due to critical soft tissue conditions. In 2 patients (0.6%), a non-displaced, stable fibular fracture was left unfixed. An additional syndesmotic positioning screw was placed in 65 cases (21.7%). Maisonneuve fractures were seen in 5 cases treated with 2 syndesmotic positioning screws via a small anterolateral approach to the distal fibula. In 166 cases (55.3%), the surgery was performed by a fellow/consultant, in 40 cases (13.4%) by a resident and in 94 cases (31.3%) by an attending physician.

The following relevant comorbidities were noted: arterial hypertension (43.7%; *n* = 131), diabetes mellitus (DM) (14.0%; *n* = 42), thereof insulin-dependent (3.7%; *n* = 11), peripheral vascular disease (0.7%; *n* = 2), osteoporosis (12.0%; *n* = 36), dementia (1.0%; *n* = 3), and rheumatoid arthritis (2.0%; *n* = 6). Furthermore, nicotine consumption was recorded in 7.3% (*n* = 22) and alcohol abuse in 4.0% (*n* = 12).

Statistical analysis was carried out with the statistics program SPSS for Windows Version 26 (SPSS Inc., Chicago, Illinois, USA). The mean values, standard deviations, minimum, maximum, median and frequencies were calculated for the collected data. The significance analysis was performed using the chi-square test, the Mann–Whitney-U test and the Kruskal–Wallis test for non-parametric data. The odds ratio and confidence interval were calculated. Multivariate analysis was conducted to identify independent risk factors for complications. The significance level was set at *p* < 0.05.

## Results

### Overall complication rates

Complications during hospital stay occurred in 41 patients (13.7%). Five patients had more than one complication. Delayed wound healing occurred in 15 patients (5%). All responded to local wound care.

Revision surgeries had to be performed in 20 cases (6.7%). Infections and wound edge necrosis requiring revision occurred in 7 (2.3%) patients, 3 of whom had deep infection necessitating hardware removal and debridement. Secondary ankle fusion was finally performed in 2 of these patients.

There were 13 patients (4.3%) with mechanical complications requiring revision. In 5 cases, postoperative CT showed fibular malalignment that was corrected with early revision. Due to preliminary weight-bearing, one patient with dementia required revision of the syndesmotic screw. Failure of internal fixation occurred in 2 patients (one with alcohol abuse and one with dementia) that were salvaged with ankle and subtalar joint fusion using a retrograde nail. In 2 cases, late syndesmotic instability developed at 2 years warranting ligamentoplasty. In 2 patients with symptomatic osteoarthritis, ankle fusion was performed after 2 years. Thus, the overall secondary fusion rate was 2.0% (6/300).

### Patient-related factors

The influence of patient demographics on the occurrence of complications is summarized in Table 1. Age was significantly associated with delayed wound healing (*p* = 0.014) and infection requiring revision (*p* = 0.008). A high BMI was correlated with delayed wound healing (*p* = 0.001) and a trend toward more infections and wound necrosis requiring revision (*p* = 0.057).

**Table 1:** The influence of patient demographics on the occurrence of complications

Patient characteristics		*n* (%)	Delayed wound healing	No delayed wound healing	Infection and wound necrosis requiring revision	No infection and wound necrosis requiring revision	Malalignment requiring revision	No malalignment requiring revision	Sensory disorder	No sensory disorder
			15 (5%)	285 (95%)	7 (2.3%)	293 (97.7%)	13 (4.3%)	287 (95.7%)	11 (3.7%)	289 (96.3%)
Age		300 (100%)	67.07 (SD:9.82)	56.23 (SD:16.54)	71.71 (SD:11.81)	56.41 (SD:16.37)	52.00 (SD:19.61)	56.99 (SD:16.27)	55.00 (SD:10.00)	56.84 (SD:16.63)
*p*-value			**0.014**		**0.008**		0.361		0.452	
BMI		166 (55.3%)	36.04 (SD:10.09)	26.54 (5.25)	36.44 (SD:12.52)	26.94 (SD:5.80)	25.04 (SD:10.02)	27.25 (SD:5.73)	26.61 (SD:6.28)	27.14 (SD:6.06)
*p*-value			**0.001**		0.057		0.924		0.673	

	*n* (%)	No complications	Delayed wound healing	Infection and wound necrosis requiring revision	Malalignment requiring revision	Sensory disorder
Sex		259 (86.3%)	15 (5%)	7 (2.3%)	13 (4.3%)	11 (3.7%)
Men	91 (30.3%)	78 (85.7%)	4 (4.4%)	2 (2.2%)	6 (6.6%)	3 (3.3%)
Women	209 (69.7%)	181 (86.6%)	11 (5.3%)	5 (2.4%)	7 (3.3%)	8 (3.8%)
*p*-value		0.837	0.751	0.918	0.205	0.822
OR/CI		1.08 (0.53, 2.19)	0.83 (0.27, 2.67)	0.92 (0.18, 4.82)	2.04 (0.67, 6.24)	0.86 (0.22, 3.31)

Significant differences are printed in bold

The impact of comorbidities on the occurrence of complications is summarized in Table 2. The presence of DM was correlated with delayed wound healing (OR 6.25) and infection or wound necrosis requiring revision (OR 4.89). Patients with insulin-dependent DM displayed a significantly higher rate of delayed wound healing (OR 37.3) and infection or wound edge necrosis requiring revision (OR 26.7) than patients without DM (*p* < 0.001).

**Table 2:** The impact of comorbidities on the occurrence of complications

		*n* (%)	No complications	Delayed wound healing	Infection and wound necrosis requiring revision	Malalignment requiring revision	Sensory disorder
Comorbitities			259 (86.3%)	15 (5%)	7 (2.3%)	13 (4.3%)	11 (3.7%)
Arterial hypertension	Yes	131 (43.7%)	105 (80.2%)	13 (9.9%)	5 (3.8%)	6 (4.6%)	6 (4.6%)
	No	169 (56.3%)	154 (91.1%)	2 (1.2%)	2 (1.2%)!!	7 (4.1%)	5 (3.0%)
*p*-value			**0.006**	**0.001**	0.134	0.853	0.459
OR/CI			2.54 (1.23, 5.03)	9.92 (2.04, 41.53)	3.31 (0.63, 17.36)	1.11 (0.36, 3.39)	1.57 (0.47, 5.28)
Diabetes mellitus (DM)	Yes	42 (14%)	30 (71.4%)	7 (16.7%)	3 (7.1%)	3 (7.1%)	3 (7.1%)
	No	258 (86%)	229 (88.8%)	8 (3.1%)	4 (1.6%)	10 (3.9%)	8 (3.1%)
*p*-value			**0.002**	** <0.001**	**0.026**	0.335	0.196
OR/CI			3.16 (1.46, 6.84)	6.25 (2.14, 18.30)	4.89 (1.10, 22.66)	1.91 (0.50, 7.24)	2.40 (0.61, 9.45)
DM (Insulin-dependent)	Yes	11 (3.7%)	5 (45.5%)	6 (54.5%)	3 (27.3%)	1 (9.1%)	0 (0%)
	No	289 (96.3%)	254 (87.9%)	9 (3.1%)	4 (1.4%)	12 (4.2%)	11 (3.8%)
*p*-value			** <0.001**	** <0.001**	** <0.001**	0.430	0.510
OR/CI			8.71 (2.52, 30.04)	37.33 (9.58, 145.43)	26.72 (5.11, 139.63)	2.31 (0.27, 19.53)	0.96 (0.94, 0.98)
Peripheral vascular disease	Yes	2 (0.7%)	1 (50%)	1 (50%)	0 (0%)	1 (50%)	0 (0%)
	No	298 (99.3%)	258 (86.6%)	14 (4.7%)	7 (2.3%)	12 (4.0%)	11 (3.7%)
*p*-value			0.133	**0.003**	0.826	**0.001**	0.782
OR/CI			6.45 (0.40, 105.2)	20.29 (1.21, 341.44)	0.99 (0.98, 1.00)	23.83 (1.41, 404.41)	0.96 (0.94, 0.99)
Osteoporosis	Yes	36 (12%)	31 (86.1%)	2 (5.6%)	3 (8.3%)	0 (0%)	2 (5.6%)
	No	264 (88%)	228 (86.4%)	13 (4.9%)	4 (1.5%)	13 (4.9%)	9 (3.4%)
*p*-value			0.967	0.870	**0.011**	0.173	0.520
OR/CI			1.02 (0.37, 2.80)	1.14 (0.25, 5.25)	5.91 (1.27, 27.57)	0.95 (0.93, 0.98)	1.67 (0.35, 8.04)
Dementia	Yes	3 (1%)	1 (33.3%)	0 (0%)	2 (66.7%)	0 (0%)	0 (0%)
	No	297 (99%)	258 (86.9%)	15 (5.1%)	5 (1.7%)	13 (4.4%)	11 (3.7%)
*p*-value			**0.007**	0.690	** <0.001**	0.711	0.734
OR/CI			13.23 (1.17, 149.38)	0.95 (0.93, 0.98)	116.8 (9.05, 1507.97)	0.96 (0.93, 0.98)	0.96 (0.94, 0.99)
Rheumatoid arthritis	Yes	6 (2%)	6 (100%)	0 (0%)	0 (0%)	0 (0%)	0 (0%)
	No	294 (98%)	253 (86.1%)	15 (5.1%)	7 (2.4%)	13 (4.4%)	11 (3.7%)
*p*-value			0.325	0.570	0.702	0.598	0.629
OR/CI			0.86 (0.82, 0.90)	0.95 (0.92, 0.97)	0.98 (0.96, 0.99)	0.96 (0.93, 0.98)	0.96 (0.94, 0.99)
Nicotine consumption	Yes	22 (7.3%)	16 (72.7%)	2 (9.1%)	1 (4.5%)	1 (4.5%)	2 (9.1%)
	No	278 (92.7%)	243 (87.4%)	13 (4.7%)	6 (2.2%)	12 (4.3%)	9 (3.2%)
*p*-value			0.054	0.360	0.475	0.960	0.160
OR/CI			2.60 (0.96, 7.10)	2.04 (0.43, 9.67)	2.16 (0.25, 18.78)	1.06 (0.13, 8.52)	2.99 (0.61, 14.78)
Alcohol abuse	Yes	12 (4%)	8 (66.7%)	0 (0%)	2 (16.7%)	2 (16.7%)	0 (0%)
	No	288 (96%)	251 (87.2%)	15 (5.2%)	5 (1.7%)	11 (3.8%)	11 (3.8%)
*p*-value			**0.043**	0.417	**0.001**	**0.032**	0.490
OR/CI			3.40 (0.97, 11.83)	0.95 (0.92, 0.97)	11.32 (1.94, 65.60)	5.04 (0.98, 25.79)	0.96 (0.94, 0.98)

Significant differences are printed in bold

Patients with peripheral vascular disease were at significantly increased risk of delayed wound healing (OR 20.3) and mechanical problems requiring revision (OR 23.8). Patients with arterial hypertension were at significantly increased risk of delayed wound healing (OR 9.92). Patients with osteoporosis had a significantly increased risk of infections and wound necrosis requiring revision (OR 5.91).

Alcohol abuse was associated with a significantly higher overall complication rate (OR 3.40), infection or wound necrosis requiring revision (OR 11.32) and mechanical problems requiring revision (OR 5.04). Two of the 3 patients with dementia required revision for wound infection or failure of internal fixation.

Complications related to fracture morphology are summarized in Table 3. The presence of an open fracture was associated with a significantly increased rate of infection and wound necrosis requiring revision (OR 14.25). Fracture-dislocation did not increase the risk of complications.

**Table 3:** Complications related to fracture morphology

		*n* (%)	No complications	Delayed wound healing	Infection and wound necrosis requiring revision	Malalignment requiring revision	Sensory disorder
Fracture morphology			259 (86.3%)	15 (5%)	7 (2.3%)	13 (4.3%)	11 (3.7%)
Open fracture		10 (3.3%)	8 (80.0%)	0 (0%)	2 (20.0%)	0 (0%)	0 (0%)
Closed fracture		290 (96.7%)	251 (86.6%)	15 (5.2%)	5 (1.7%)	13 (4.5%)	11 (3.8%)
*p*-value			0.553	0.461	** <0.001**	0.494	0.530
OR/CI			1.61 (0.33, 7.86)	0.95 (0.92, 0.97)	14.25 (2.39, 84.84)	0.96 (0.93, 0.98)	0.96 (0.94, 0.98)
Dislocation	Yes	107 (35.7%)	90 (84.1%)	7 (6.5%)	3 (2.8%)	5 (4.7%)	5 (4.7%)
	No	193 (64.3%)	169 (87.6%)	8 (4.1%)	4 (2.1%)	8 (4.1%)	6 (3.1%)
*p*-value			0.404	0.362	0.688	0.830	0.490
OR/CI			1.33 (0.68, 2.60)	1.62 (0.57, 4.59)	1.36 (0.30, 6.21)	1.13 (0.36, 3.56)	1.53 (0.46, 5.13)

Significant differences are printed in bold

### Surgery-related factors

Complications related to surgical treatment are listed in Table 4. Staged treatment with primary external fixation was associated with a significantly increased risk of delayed wound healing compared to primary internal fixation (OR 3.14).

**Table 4:** Complications related to surgical treatment

	*n* (%)	No complications	Delayed wound healing	Infection and wound necrosis requiring revision	Malalignment requiring revision	Sensory disorder
		259 (86.3%)	15 (5%)	7 (2.3%)	13 (4.3%)	11 (3.7%)
Single surgery	156 (52%)	138 (88.5%)	4 (2.6%)	4 (2.8%)	5 (3.2%)	4 (2.8%)
Staged treatment	144 (48%)	121 (84.0%)	11 (7.6%)	3 (1.9%)	8 (5.6%)	7 (4.5%)
*p*-value		0.264	**0.044**	0.624	0.318	0.431
OR/CI		1.46 (0.75, 2.83)	3.14 (0.98, 10.10)	1.46 (0.32, 6.63)	1.78 (0.57, 5.56)	0.61 (0.17, 2.12)
Syndesmotic screw	65 (21.7%)	56 (86.2%)	4 (6.2%)	1 (1.5%)	6 (9.2%)	1 (1.5%)
No syndesmotic screw	235 (78.3%)	202 (86.3%)	11 (4.7%)	6 (2.6%)	7 (3.0%)	10 (4.3%)
*p*-value		0.972	0.635	0.629	**0.029**	0.300
OR/CI		1.02 (0.46, 2.25)	1.33 (0.41, 4.32)	0.59 (0.07, 5.02)	3.30 (1.07, 10.18)	0.35 (0.04, 2.79)
Surgical experience						
Resident	40 (13.3%)	35 (87.5%)	0 (0%)	1 (2.5%)	2 (5.0%)	2 (5.0%)
Fellow/Consultant	166 55.3%)	143 (86.1%)	10 (6%)	4 (2.4%)	8 (4.8%)	6 (3.6%)
Attending physician	94 (31.3%)	81 (86.2%)	5 (5.3%)	2 (2.1%)	3 (3.2%)	3 (3.2%)
*p*-value		0.974	0.288	0.987	0.805	0.877

Approach to fibular fracture	*n* (%)	No delayed wound healing	Delayed wound healing
		285 (95%)	15 (5%)
No treatment	6 (2%)	6 (100%)	0 (0%)
Lateral approach	225 75.3%)	214 (95.1%)	11 (4.9%)
Posterolateral approach	65 (21.7%)	64 (98.5%)	1 (1.5%)
Lateral stab incision	3 (1%)	3 (100%)	0 (0%)
*p*-value		0.295	

Approach to PM fracture	*n* (%)	No delayed wound healing	Delayed wound healing
		285 (95%)	15 (5%)
No treatment	139 (46.3%)	139 (100%)	0 (0%)
Anterior approach	38 (12.7%)	38 (100%)	0 (0%)
Posterolateral approach	109 (36.3%)	108 (99.1%)	1 (0.9%)
Posteromedial approach	8 (2.7%)	8 (100%)	0 (0%)
Medial approach	6 (2%)	5 (83.3%)	1 (16.7%)
*p*-value		** <0.001**	

Significant differences are printed in bold

Malalignment requiring revision was detected in 9.2% of cases after syndesmotic screw placement compared with 3% in patients without (OR 3.30; *p* = 0.029). In 4 of 5 cases requiring revision for postoperative malalignment detected with CT, no fixation of the PM fragment had been performed initially.

In 11 of 12 cases with delayed wound healing, a lateral approach to the fibula fracture was used and in one case a posterolateral approach had been used for both fibular and PM fracture fixation. The rates of delayed wound healing were 4.9% (11 of 225) following a lateral approach and 1.5% (1 of 65) following a posterolateral approach (*p* = 0.295). Delayed wound healing occurred in 2 approaches used exclusively for PM fixation: in 1 of 109 (0.9%) posterolateral approaches, and in 1 of 6 (16.7%) medial approaches. Infections requiring revision occurred in 3 out of 225 (1.3%) lateral approaches and in 1 out of 6 (16.7%) medial approaches.

Among 100 patients from the whole cohort who had a physical follow-up examination at our hospital for another study [10], a sensory deficit was found in 11 patients. The superficial peroneal nerve was affected permanently in 2 patients, and temporarily in another 2. Two patients reported hyposensitivity over the scar of the posterolateral approach and 3 patients over the scar of the medial approach. Hyposensitivity over the heel occurred temporarily in 2 patients and permanently in one. None of the 100 patients had signs of a sural nerve affection.

Multivariate analysis identified BMI (*p* = 0.028), insulin-dependent DM (*p* = 0.003), and staged fixation (*p* = 0.043) as independent risk factors for delayed wound healing. Peripheral vascular disease (*p* = 0.040), alcohol abuse (*p* = 0.033), and use of syndesmotic positioning screw (*p* = 0.044) were found to be independent risk factors for mechanical problems requiring revision.

## Discussion

The treatment of ankle fractures involving the PM has undergone considerable changes over the past decade [4–13]. The aim of our study was to identify risk factors for complications following surgical treatment in a sizeable number of patients.

Complications occurred in 13.7% of cases with superficial wound healing problems that resolved without further intervention being the most prevalent one. The overall infection rate in our study of 2% compares well with the data from the current literature ranging from 1.44 to 14.0% [14, 15, 19–26]. The secondary ankle fusion rate of 2% at an average follow-up of 9 years is only slightly higher compared to the numbers in the literature, which range from 0.44 to 0.96% but with shorter follow-up [22, 25, 27].

### Patient-related risk factors

We identified several significant patient-related risk factors for infections, above all comorbidities like insulin-dependent DM, peripheral vascular disease, alcohol abuse and dementia. The association of DM with infection following ankle fracture fixation is well established [28]. Furthermore, a high BMI increased the risk of infections and delayed wound healing as also reported in previous studies [15, 20, 21, 23].

Our data showed a correlation between age and wound complications, as also described in earlier studies [15, 21, 29]. Patients with a higher age display more comorbidities, which in turn are associated with more complications [26, 30, 31]. On multivariate analysis, BMI and insulin-dependent DM but not age remained independent risk factors for wound healing problems.

We also found a correlation between dementia and infection or wound necrosis requiring revision. These patients tend to be noncompliant. Rather than patient age, comorbidities seem to be decisive for the risk of complications and the postoperative protocol needs to be adapted accordingly [30, 31].

The influence of cigarette consumption in terms of complications is controversial [21, 24, 26, 27, 29, 32]. Nåsell et al. [27], in a group of 906 patients, found significantly more deep wound infections in smokers than in non-smokers. In our study, 87.4% of patients without nicotine consumption had no complications compared to only 72.7% in patients with nicotine consumption (*p* = 0.054). Similar to the investigations of Olsen et al. [21] this difference did not reach statistical significance.

Like in previous studies, open fractures constituted a significant patient-related risk factor for infections [22, 29]. Initial ankle dislocation has been associated with poor outcome [15, 33] and late posttraumatic arthritis [34]. In keeping with others [14, 15, 24, 33, 35] we did not see a correlation between fracture-dislocations and complication rates.

### Surgeon-related risk factors

Serveral studies have demonstrated that the posterolateral approach allows for a better quality of reduction of the PM fragment, particularly in the presence of smaller, depressed and intercalary fragments [5, 19, 36–40]. Biomechanically, posterior screws and antiglide plates to the PM and distal fibula provide more stability than anterior-to posterior screws or lateral plates, respectively [33, 36, 37, 41].

Pilskog et al. [38] found similar clinical results and complication rates with the anterior approach and indirect AP screw fixation compared the posterolateral approach and direct fixation of the PM. The need for a syndesmotic screw was significantly reduced with the latter. The reoperation rate (7%) was almost identical to our results (6.7%). Similar low complication rates with the posterolateral approach were reported by others [39, 40, 42].

On the other hand, Pinho-Tavares et al. [13] reported 44% (19/43) delayed wound healing with the posterolateral approach and referred to a considerable learning curve. Mertens et al. [12] saw a hallux flexion deficit in 30% and sural nerve lesions in even 38% of 50 patients treated via a posterolateral approach. In contrast, we did not see infections nor sural nerve lesions following the posterolateral approach. Malalignment occurred more frequently if the PM fragment was not fixed (2.9%) compared with 0.9% of PM fragments fixed via a posterolateral approach. Sensory deficits were only observed with the small anterior approach for indirect PM fixation. Meticulous soft tissue handling including identification and protection of the sural nerve is a prerequisite for avoiding complications.

The majority of the distal fibular fractures in the presence of a PM fracture can be easily reduced and fixed with a posterior antiglide plate using the same posterolateral approach [6, 17, 37]. In accordance with others [25, 40–42], we found a low overall complication rate with the posterolateral approach (1.5 vs. 4.9% with the lateral approach) none of which required revision.

Staged fixation, that was identified as a negative prognostic factor with respect to functional outcome in our previous study [10], also was an independent risk factor for complications. This most likely reflects the severity of injury, as this is employed in highly unstable fracture-dislocations or with critical soft tissue conditions [4].

Our study has several limitations. Patient data were retrieved retrospectively from the files. Therefore, data like sensory disorders, late ankle fusion, nicotine consumption, or alcohol abuse, may be underreported. Long-term sequelae like posttraumatic arthritis and functional deficits could not be investigated. On the other hand, to the best of our knowledge, we report the largest patient cohort with respect to complications following malleolar fractures involving the PM.

In conclusion, we identified significant risk factors for the occurrence of complications following PM fractures. Treatment should be tailored to the individual pathoanatomy and known risk factors. Fixation of the fibular fracture and the PM fragment via a common posterolateral approach is associated with minimal morbidity.
